# In Situ Formation of Calcium Zirconate Particles on the Surface of High-Translucent Zirconia: A New Way to Strongly Improve Its Bonding Properties

**DOI:** 10.3390/jfb17050227

**Published:** 2026-05-06

**Authors:** Zhen Yang, Yueming Tian, Jianguo Tan, Ti Zhou, Xuedong Wang, Xinshu Dong, Mingyue Liu, Yanheng Zhou

**Affiliations:** 1 Department of Prosthodontics, Peking University School and Hospital of Stomatology, Beijing 100081, China; bdkqyangzhen@bjmu.edu.cn (Z.Y.); kqtanjg@bjmu.edu.cn (J.T.); 1910303136@pku.edu.cn (X.D.); 2 National Center of Stomatology, Beijing 100081, China; 3 National Clinical Research Center for Oral Diseases, Beijing 100081, China; 4 National Engineering Research Center of Oral Biomaterials and Digital Medical Devices, Beijing 100081, China; 5 Beijing Key Laboratory of Digital Stomatology, Beijing 100081, China; 6 Beijing Key Laboratory of Biomaterials for Oral Diseases, Beijing 100081, China; 7 Beijing Key Laboratory for Intelligent Biomanufacturing and Regeneration of Craniofacial Tissues, Beijing 100081, China; 8 NHC Key Laboratory of Digital Stomatology, Beijing 100081, China; 9 NMPA Key Laboratory for Dental Materials, Beijing 100081, China; 10 Center of Stomatology, Beijing Tsinghua Changgung Hospital, School of Clinical Medicine, Tsinghua Medicine, Tsinghua University, Beijing 102218, China; tyma05562@btch.edu.cn (Y.T.); wang-xuedong@tsinghua.edu.cn (X.W.); 11 Department of Dental Esthetics, Binzhou Medical University Affiliated Yantai Stomatological Hospital, Yantai 264000, China; zhoutiemail@163.com; 12 First Clinical Division, Peking University School and Hospital of Stomatology, Beijing 100081, China

**Keywords:** high-translucency zirconia, surface modification, MDP, bonding, thermal cycling aging, chemical characterization

## Abstract

High-translucency zirconia (HTZ) has superior esthetic properties, but its unreliable resin bonding limits minimally invasive anterior restorations. An in situ surface modification was developed to synthesize CaZrO_3_ particulates on pre-sintered HTZ for enhanced bonding durability. HTZ specimens were randomized into control (Zr-c) and calcium-modified (Zr-Ca) groups; Zr-Ca was treated with NaF/HCl mixture, calcium chloride glycerol solution, NaOH incubation (80 °C, 2 h), and sintering. Surface characteristics were characterized by SEM/EDS, AFM, X-ray diffraction (XRD), X-ray photoelectron spectroscopy (XPS), and FTIR. Flexural strength was tested via three-point bending; shear bond strength (SBS) was evaluated immediately and after 5000 thermocycles with resin cements (with/without 10-MDP). Zr-Ca showed uniform surface particulates, increased roughness, enhanced wettability, and surface Ca; XRD/FTIR/XPS confirmed CaZrO_3_ and Ca-O-P species (after MDP). Zr-Ca with 10-MDP-containing resin adhesive had significantly higher SBS before/after aging (predominantly mixed failures), with flexural strength within clinical limits. In situ CaZrO_3_ formation on HTZ strengthens MDP-mediated resin bonding and thermocycling resistance while preserving mechanical integrity, providing a feasible strategy for durable adhesion.

## 1. Introduction

Zirconia-based restorations have been widely utilized in dentistry due to their excellent mechanical and biocompatible properties. Conventional yttria-stabilized tetragonal zirconia polycrystal (3Y-TZP) exhibits high fracture toughness and flexural strength, which is conducive to the preservation of the remaining tooth structure by fabricating full-contour, monolithic zirconia restorations. However, its esthetic properties, particularly translucency, remain inferior compared to silica-based ceramics, which is a major limitation that affects its clinical application in the anterior esthetic zone. To address this issue, the latest iteration of zirconia employs a higher percentage of Y_2_O_3_-stabilizer (≥4 mol%) and an increased content of cubic crystalline phases, enhancing the transparency of zirconia to meet esthetic requirements [1]. Even though the flexural strength of this high-translucency zirconia (HTZ) decreases significantly (550–800 MPa), compared with that of conventional 3Y-TZP (900–1400 MPa), it still surpasses that of silica-based ceramics [2]. Due to superior optical properties and mechanical strength, the clinical applications of HTZ have greatly expanded, with researchers recently exploring its use in anterior minimally invasive restorations, e.g., porcelain laminate veneers [3].

When HTZ is applied for minimally invasive restorations where bonding retention is the main way of retention, a strong and durable bond is a fundamental prerequisite for long-term success. Nevertheless, similar to 3Y-TZP, HTZ is a chemically inert ceramic due to its compact crystal structure and the lack of silica phase. The conventional bonding protocols established for silica-based ceramics (i.e., acid etching followed by the application of silane coupling agent) do not work effectively with zirconia [4,5]. Thus, to improve its surface properties and bonding receptiveness, both mechanical and chemical surface conditioning methods have been investigated, such as air-borne particle abrasion [6], selective infiltration etching [7], hot chemical etching [8], Er:YAG, Nd:YAG, or CO_2_ laser irradiation [9], gas plasma treatment [10], nano-structured alumina coating [11], silica coating [12,13], deposition of low-fusing porcelain pearl layer [14], applying phosphate acidic/functional silane monomers [15], among others. However, current surface treatments have some drawbacks: micromechanical-based protocols such as air-borne particle abrasion may lead to phase transformation and microcracks, which reduce the functional strength of zirconia; severe coating separation may occur in various coatings due to mismatched thermal expansion coefficients. In addition, these studies found less favorable bonding performance than for silicate-based ceramics [16]. Moreover, the major purpose of these surface treatments is to promote the bond strength of conventional 3Y-TZP, while research reporting on their influences on HTZ is relatively scarce. According to Ruales-Carrera et al., HTZ exhibited lower bond strength with resin cements than conventional 3Y-TZP subjected to the same treatment procedures [17]. However, when the application of HTZ is extended to minimally invasive partial-coverage restorations where preparation designs provide limited or no mechanical retention, the requirements for bonding are much higher. Therefore, there is a strong need to further improve the bonding performance of HTZ.

Surface treatments are generally applied after the sintering process of zirconia. However, applying pre-treatments in different sintering stages may lead to markedly different surface textures [18]. Treating the pre-sintered zirconia specimens has been reported to provide rougher surfaces and higher resin cement bond strength when compared to post-sintering application [19]. Our research group developed a method of in situ-synthesized polycrystalline particulates (ZrO_2_) on the surface of undensely sintered 3Y-TZP. By enhancing surface roughness, the bonding properties of zirconia were effectively improved without significantly decreasing its mechanical properties [20,21]. The present study aims to further increase the chemical bonding by combining with 10-methacryloyloxydecyl dihydrogen phosphate (10-MDP). Pre-treatment agents containing functional monomer 10-MDP are one of the most widely used protocols for modifying the surface of zirconia prior to cementation [22,23,24]. On the other hand, 10-MDP-containing dental adhesives are well known to effectively improve the bond strengths of adhesives to dentin, thanks to the chemical bond provided by 10-MDP, which contains two terminal P-OH groups that react with Ca hydrolyzed from dentin to generate stable 10-MDP-Ca salts [25]. Inspired by these investigations, the present study proposes to in situ synthesize calcium zirconate (CaZrO_3_) particulates on the surface of HTZ, not only to enhance the mechanical locking with resin cement by roughening the surface of zirconia, but also to promote the formation of chemical bonds (MDP-Ca) between CaZrO_3_ and 10-MDP, ultimately significantly enhancing the bonding strength of HTZ.

**Figure 1:**
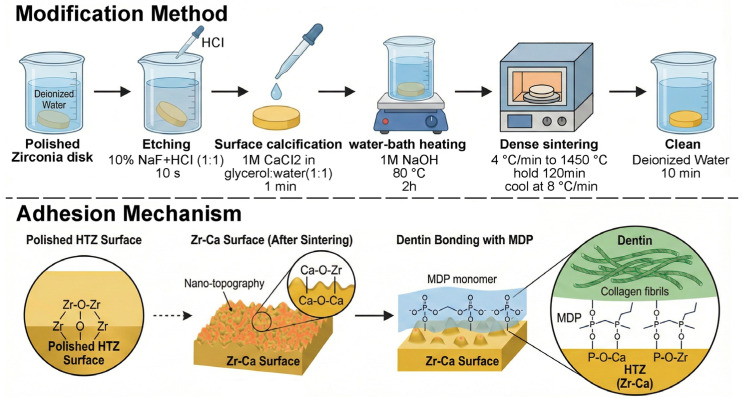
Modification method and adhesion mechanism schematic diagram of this study.

The purpose of this study was to explore the potential of the in situ synthesis of CaZrO_3_ particulates to improve the bond strength of the resin cement/HTZ interface. Physical and chemical alterations in the HTZ were assessed after surface treatment, as were the immediate shear bond strength (SBS) and SBS after aging. The null hypothesis was that the in situ-synthesized particulates would have no influence on the bonding between HTZ and resin cement. The overall schematic diagram of this study is shown in Figure 1.

## 2. Materials and Methods

### 2.1. Specimen Preparation and Treatments

Prefabricated high-translucent zirconia disks (20 mm diameter, 2 mm thickness, Upcera Dental Technology Co., Ltd., Shenzhen, China) and zirconia bars (25 × 5 × 1.5 mm, Upcera Dental Technology Co., Ltd., China) were fabricated. The surface of the sample was polished with 1200-grit SiC abrasive. The substrates were ultrasonically cleaned with deionized water to remove surface debris before use. The specimens were randomly divided into 2 groups: Zr-c as the control and Zr-Ca as the experiment group. The experiment groups were immersed into 10% sodium fluoride and hydrochloric acid solution (NaF:HCl = 1:1) for 10 s, then they were dipped into a 1 M calcium chloride glycerol solution (glycerin:water = 1:1) on the upper surface and left for 1 min. After that, treated specimens were put into a 1 M NaOH solution and held at 80 °C for 2 h. Afterward, all the specimens were sintered in the sintering furnace (ZSK 1700, Cassini (Beijing) Electric Co., Ltd., Beijing, China), with the heating speed of 4 °C/min to 1450 °C. After 120 min at 1450 °C, all samples were naturally cooled at 8 °C/min to room temperature, and then cleaned in deionized water for 10 min prior to use.

### 2.2. Surface Characterization

A scanning electron microscope (Phenom-world Co., Ltd., Eindhoven, The Netherlands, SEM) was used to evaluate the surface morphology of the zirconia before and after the treatment. The composition of zirconia surfaces was investigated with energy dispersive spectrometry (EDS).

Surface topography was observed by White Light Interferometer (ContourX-100, Bruker, Billerica, MA, USA, WLI), and peak-to-valley surface roughness (Ra) was determined. Three samples per group were observed in five random fields per sample.

The surface wettability of each specimen was characterized using an automatic contact angle meter (OCA15Pro, DataPhysics Instruments GmbH, Filderstadt, Germany). A 0.5 μL droplet of ultrapure water was placed on each surface, and the static contact angle was determined from the captured sessile drop image as the angle between the drop profile and the projected specimen surface at the three-phase contact line.

### 2.3. Surface and Structural Chemical Characterization

The surface composition of Zr-Ca specimens with/without MDP was analyzed by X-ray Photoelectron Spectroscopy (ESCALAB 250, Thermo Fisher Scientific, Waltham, MA, USA) using monochromatic Al Kα (hν = 1486.6 eV). Binding energies were charge-referenced to C 1 s = 284.8 eV; Shirley backgrounds and Scofield sensitivity factors were used to quantify compositions; high-resolution peaks were fit with mixed Gaussian–Lorentzian line shapes.

X-ray diffraction (XRD) of Zr-c and Zr-Ca was carried out on an XRD-7000 diffractometer (Shimadzu, Kyoto, Japan) using Cu Kα radiation (λ = 1.5406 Å; Ni Kβ filter; 40 kV, 30 mA). To probe near-surface crystal phases of zirconia, a grazing-incidence geometry was used with a fixed incidence angle α = 1.0° and sample spinning (~30 rpm). Diffraction patterns were collected from 20 to 100° 2θ with a step size of 0.02°. Raw data were background-subtracted and Kα2-stripped prior to analysis. Phase identification was performed by matching peak positions/intensities to ICDD PDF-4+ entries for monoclinic, tetragonal, and cubic ZrO_2_. The monoclinic fraction was additionally estimated by the Toraya modification of the Garvie–Nicholson method using the m(–111), m(111), and t(101) reflections as a cross-check.

Attenuated Total Reflectance–Fourier Transform Infrared Spectroscopy (ATR-FTIR) of Zr-Ca specimens with/without MDP and CaZrO_3_ was recorded on a Bruker ALPHA spectrometer equipped with a diamond ATR accessory. Spectra (4000–400 cm^−1^, 4 cm^−1^ resolution) were averages of 32 co-added scans after an air background; ATR correction and baseline subtraction were applied as needed.

### 2.4. Three-Point Bending Tests

Zirconia bars of Zr-c and Zr-Ca groups were used for three-point bending tests. The tests were performed on a universal force testing machine at a crosshead speed of 0.5 mm/min until the fracture of the specimens. The average three-point bending strength was calculated based on the 10 data points of each group. The formula to calculate the three-point bending strength was as follows:

σ = 3Fl/2wb^2^ (1)

F is the breaking load (N), l represents the test span (mm), w is the width of the specimen (mm), and b is the thickness of the specimen (mm); the calculation result σ is the three-point bending strength (MPa).

### 2.5. Shear Bonding Strength Test (SBS Test)

The specimens of the Zr-c group and Zr-Ca were divided into two subgroups, respectively, as Zr-c1, Zr-c2, and Zr-Ca1, Zr-Ca2. All disks were ultrasonically cleaned in deionized water for 8 min, dried with clean air flow, and kept in a desiccator. Self-adhesive resin cement (Valux™ Plus, 3M ESPE, St. Paul, MN, USA) was properly made into resinous columns (5 mm in diameter, 10 mm in thickness) using stainless steel molds. Composite resin bonding cement (BISCO, Schaumburg, IL, USA) was properly applied to bond the zirconia specimen and resinous column. Group Zr-c1 and Zr-Ca1 were directly bonded to the resinous column with duo-link universal resin luting cement. However, the surface of group Zr-c2 and Zr-Ca2 was firstly treated with Z-prime for 20 s, dried, and then bonded to a resinous column with duo-link universal resin luting cement. After that, all the specimens were subjected to a 20 N force for 10 min, and an additional 40 s of light irradiation was applied from each side of the specimens with the LED lamp to ensure optimal polymerization. All disks were then kept at 37 °C for 24 h. Afterwards, the four groups of specimens were randomly divided into either the (1) no aging (without T, n = 10) or (2) aging group (with T, n = 10). In the aging group, samples were immersed in a water bath at 5 °C and 55 °C with a dwell time of 30 s at each temperature. The delay time between immersion in two water baths was 3 s, and 5000 thermal cycles were performed.

Shear bond tests were performed with a universal force testing machine (AG-X Plus, Shimadzu Co., Ltd., Shimadzu, Japan) at a crosshead speed of 0.5 mm/min until the separation of the resinous column from the zirconia-based ceramic. The average shear bond strength was calculated from 10 data points. Each shear bond strength data was calculated according to the following formula:

SBSσ = F/S(2)

where SBSσ is shear bond strength (MPa), F is maximum load shear force (N), and S is bonded area (mm^2^).

### 2.6. Failure Mode Analysis

The failure mode of the adhesive interface was observed using an optical microscope (SMZ-10, Nikon, Tokyo, Japan). Failure modes were classified as follows.

The failure between the zirconia and resin bonding surface was defined as ‘adhesive failure’, and the failure within either the zirconia or resin stub was defined as ‘cohesive failure’. The term ‘mixed’ failure was used to describe the combination of these two failure types. Fractured surfaces were observed using a scanning electron microscope under 1000× magnification.

### 2.7. Statistical Analysis

All results are expressed as means and standard deviations, and statistical significance was tested using one-way ANOVA. All statistical analyses were performed using SPSS software version 25.0 (SPSS, IBM, Armonk, NY, USA). The value of *p* < 0.05 was statistically significant.

## 3. Results

### 3.1. Surface Characterization

**Figure 2:**
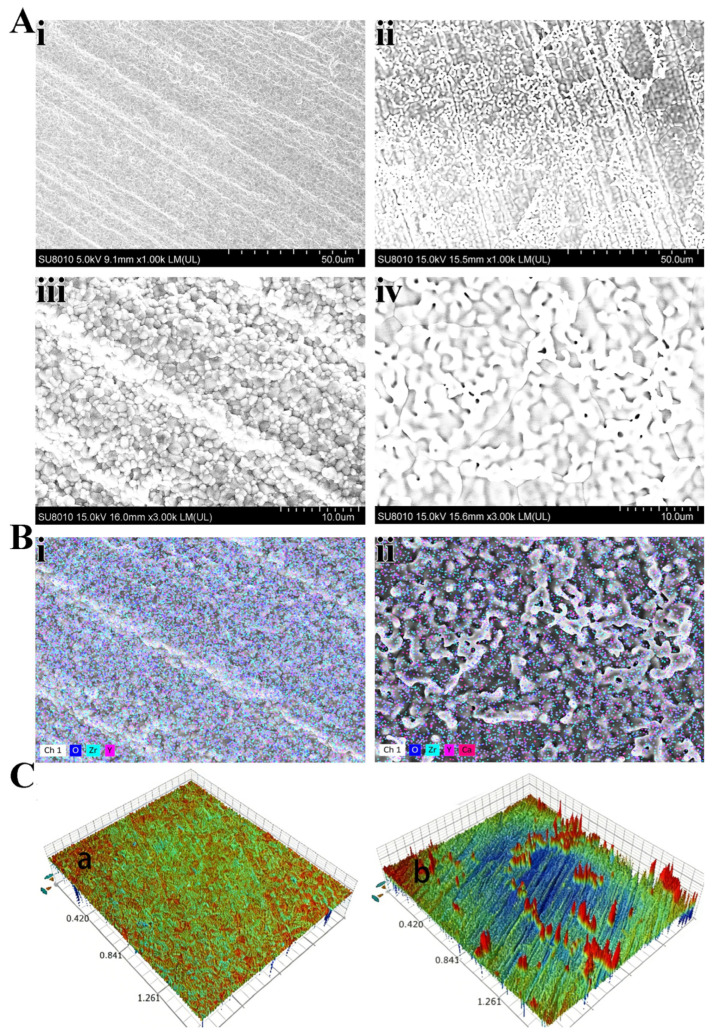
Surface morphology among groups. (**A**) Representative SEM images of surface morphology of Zr-c and Zr-Ca groups ((**i**) Zr-c group × 1000; (**ii**) Zr-Ca group × 1000; (**iii**) Zr-c group × 3000; (**iv**) Zr-Ca group × 3000). (**B**) EDS analysis of Zr-c and Zr-Ca groups. ((**i**) Zr-c group; (**ii**) Zr-Ca group) (**C**). Surface contour images of Zr-c and Zr-Ca groups ((**a**) Zr-c group; (**b**) Zr-Ca group). Scale bar = 50 μm in (**Ai**) & (**Aii**); Scale bar = 10 μm in (**Aiii**) &(**Aiv**).

The surface morphology was evaluated by SEM and the Contour Measuring Machine. The results of SEM (Figure 2A) showed that the Zr-c group had a flat surface feature (Figure 2(Ai)), while different distributions of particulates could be seen on the surface of the Zr-Ca group (Figure 2(Aii)). When magnified 3000 times, rivet particles can be better observed on the modified zirconia surface (Figure 2(Aiv)). Energy dispersive spectrometry (EDS) was used to analyze the surface characteristics of materials before and after modification (Figure 2B). Three disks of each group were selected for analysis, and the atomic content ratio of each group of samples was displayed in Table 1. The representative images were demonstrated. The results of EDS showed that the surface composition of the Zr-c group was Zr, O. After the surface treatment, the proportion of O increased, the ratio of Zr decreased, and 3.11% Ca appeared. Representative 3D images are shown in Figure 2C. It can be intuitively seen that obvious protrusions are shown on the surface of the treated zirconia.

### 3.2. Surface Roughness and Wettability

**Figure 3:**
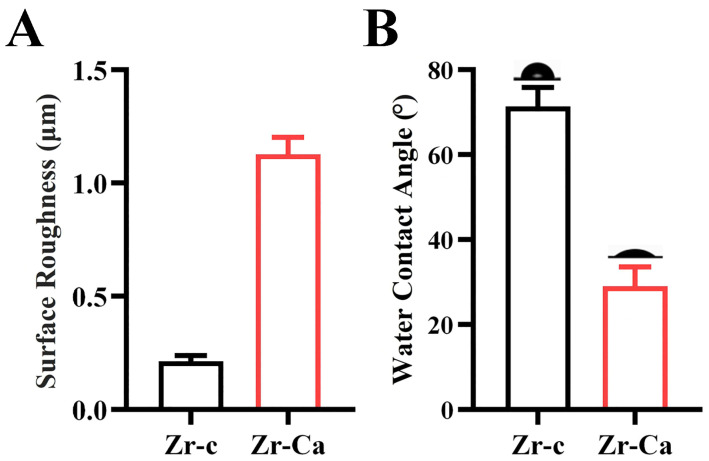
Surface roughness and wettability before and after the surface modification. (**A**) Surface roughness of Zr-c and Zr-Ca groups. (**B**) Water contact angle of Zr-c and Zr-Ca groups.

Figure 3 shows the mean and standard deviation values of surface roughness and wettability of control and experimental groups. Compared with the Zr-c group (0.21 ± 0.03 μm), Ra values of the experimental groups were 1.13 ± 0.54 μm. The surface roughness test results also showed that the arithmetic mean height of the 3D surface roughness (Sa) of the control group (Zr-c) was 0.85 ± 0.05 μm, while the Sa value of the modified experimental group (Zr-Ca) was 1.74 ± 0.19 μm. The data and statistical results are presented in the Supplementary Data. The particles on the modified zirconia surface significantly increased the surface roughness. The water contact angle measurements represent the wettability of the material surface. The high contact angle (70.5 ± 4.21°) of the water droplet on the Zr-c group demonstrated low hydrophilicity of the untreated surface. After particulates were generated on the surface, the contact angle substantially decreased to 28.6 ± 5.11°. Modification of the specimen surface increases its wettability.

### 3.3. Surface and Structural Chemical Characterization

**Figure 4:**
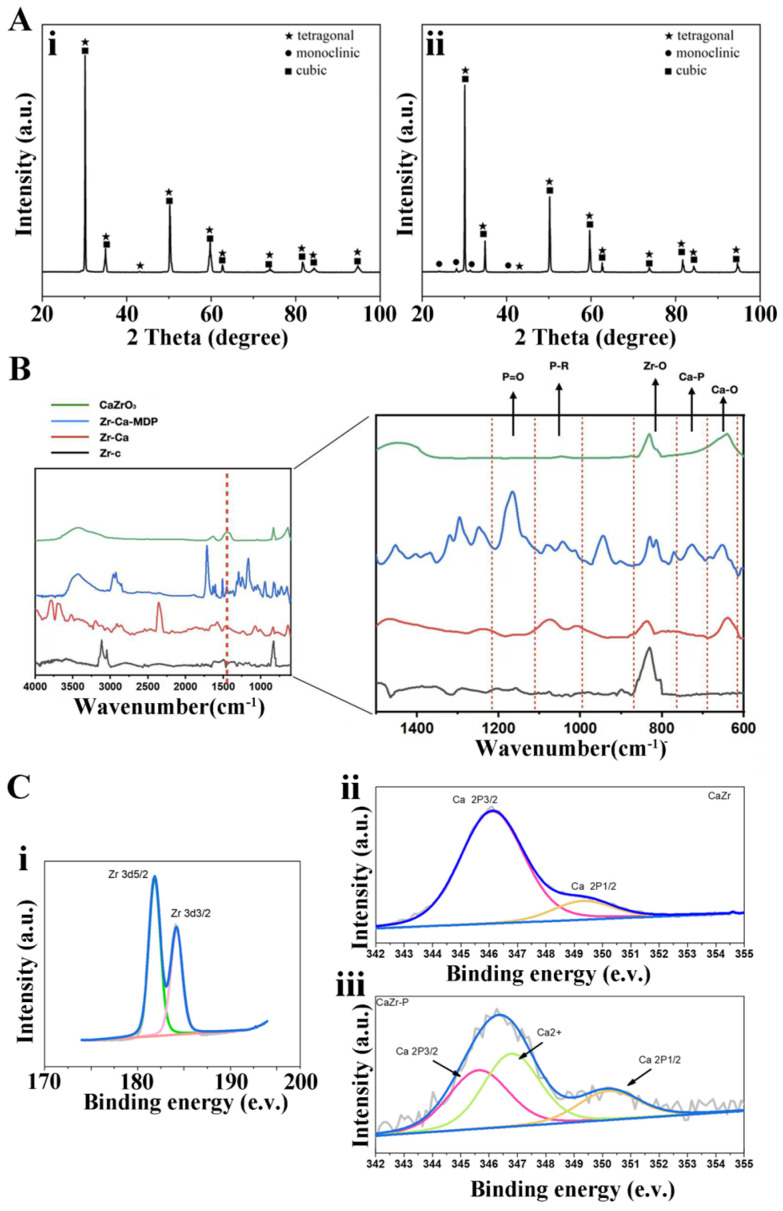
Surface and structural chemical characterization of the groups. (**A**) The composition phase of Zr-c group (**i**) and Zr-Ca group (**ii**); (**B**) chemical composition and functional group structure of Zr-c, CaZrO_3_, and Zr-Ca specimen surfaces before and after MDP coating; (**C**) elemental composition and chemical states of Zr-O (**i**), and Zr-Ca specimens surface before (**ii**) and after (**iii**) MDP coating.

XRD revealed that, relative to the Zr-c group (Figure 4(Ai)), the treated Zr-Ca specimens display additional diffraction peaks assignable to monoclinic ZrO_2_ (m-ZrO_2_) (Figure 4(Aii)). The very low integrated intensity of these reflections indicates only a minor monoclinic fraction. The emergence of m-ZrO_2_, together with the introduction of Ca, is consistent with partial replacement of the original Y^3+^ stabilizer by Ca^2+^ during treatment, which locally reduces the phase stability of zirconia.

Figure 4B shows the FTIR spectra of Zr-c, Zr-Ca, Zr-Ca-MDP, and CaZrO_3_. All samples exhibit bands in the 4000–1000 cm^−1^ range. Peak segmentation analysis was performed within the range of 1500 cm^−1^. The group of Zr-c shows Zr-O bands at 840 cm^−1^. Compared with Zr-c, the Zr-Ca spectrum displays additional bands attributable to Ca-O vibrations at 640 cm^−1^. This is the location of a characteristic peak indicating the presence of Ca on the surface. Pure high-acid calcium particles (CaZrO_3_) also show clear Zr-O and Ca-O bonds at the corresponding positions. After MDP treatment (Zr-Ca-MDP), new absorption bands appear. The presence of P-R and P=O bonds in the range of 1000 cm^−1^ to 1200 cm^−1^ confirms the presence of phosphate esters on the modified surface. The presence of Ca-P bonds at the range of 700~800 cm^−1^ provides relatively definitive confirmation that the Ca generated after surface modification forms a chemical bond with the phosphate primer.

The XPS spectra of the samples reveal characteristic Zr 3d and Ca 2p doublets arising from spin–orbit splitting (Figure 4C). The Zr 3d region shows two main peaks at ~182.0 eV (Zr 3d5/2) and ~184.4 eV (Zr 3d3/2) with a splitting of ~2.4 eV, consistent with Zr^4+^ species (Figure 4(Ci)). Deconvolution indicates two chemical environments: a higher binding-energy component attributed to ZrO_2_ and a lower one to CaZrO_3_. The Ca 2p spectra exhibit peaks at ~346.6 eV (Ca 2p3/2) and ~350.1 eV (Ca 2p1/2), confirming Ca in oxide coordination without significant carbonate contamination (Figure 4(Cii)). After MDP deposition, an additional Ca 2p component attributable to Ca^2+^ coordinated by phosphate appeared, and the relative area of the Ca 2p1/2 peak increased (Figure 4(Ciii)). These changes indicate that the phosphate groups in MDP interact with surface CaZrO_3_ to form Ca-O-P species.

### 3.4. Three-Point Bending Test

**Figure 5:**
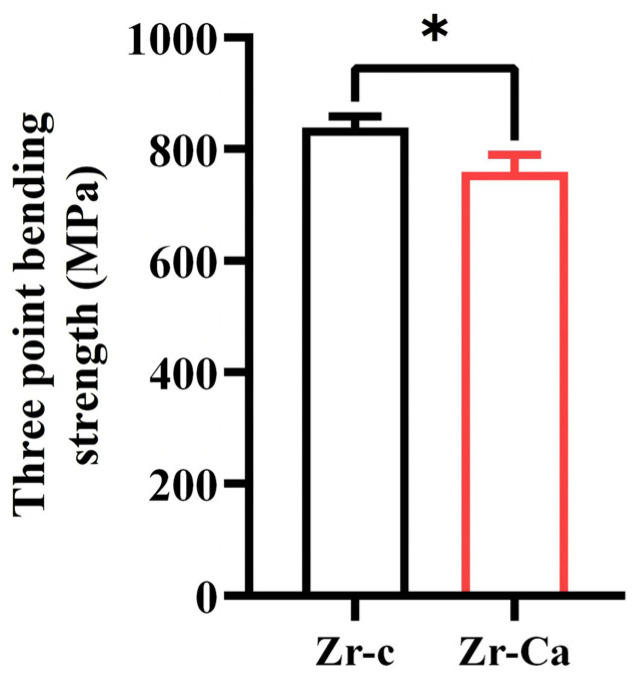
The result of the three-point bending test. *, *p* < 0.05.

Three-point bending values are shown in Figure 5. The control group had the mechanical strength of 834.12 ± 32.14 MPa. After the surface treatment, the mechanical strength decreased to 758.25 ± 24.64 MPa. Surface modification slightly reduces the flexural strength of high-translucency zirconia (*p* < 0.05); however, the strength can still meet the requirements of clinical application.

### 3.5. Shear Bond Strength (SBS) Test

**Figure 6:**
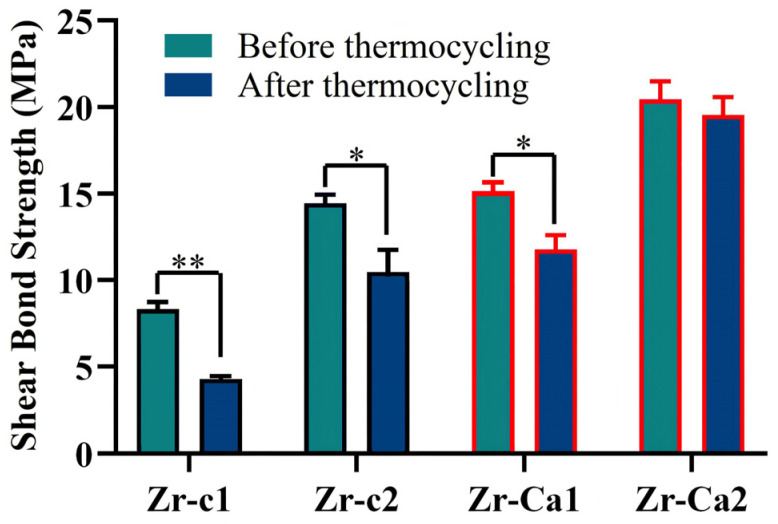
SBS of the experimental groups and the control group. Zr-c1: untreated high-translucency zirconia using resin adhesive; Zr-c2: untreated high-translucency zirconia using resin adhesive containing MDP; Zr-Ca1: surface-generated calcium zirconate particle group using resin adhesive; Zr-Ca2: surface-generated calcium zirconate particle group using resin adhesive containing MDP. *, *p* < 0.05. **, *p* < 0.01.

The results of the SBS test are shown in Figure 6. The SBS of the resin adhesive group containing MDP is significantly better than that of the simple resin adhesive group. After the surface treatment, the immediate SBS of the surface-generated calcium zirconate particle group using simple resin adhesive (Zr-Ca1, 14.68 ± 1.77 MPa) is comparable to the immediate SBS of the untreated group using MDP-containing resin adhesive (Zr-c2, 13.89 ± 1.19 MPa). Moreover, the surface-modified group containing MDP resin (Zr-Ca2) showed the highest immediate SBS (20.02 ± 2.25 MPa), twice as much as the Zr-c1 group.

After thermocycling, the SBS values of all four groups exhibited a decrease. To quantify the aging effect, the intragroup SBS variations were analyzed by comparing the thermocycled specimens with their non-aged counterparts (Zr-c1T vs. Zr-c1, Zr-c2T vs. Zr-c2, Zr-Ca1T vs. Zr-Ca1, and Zr-Ca2T vs. Zr-Ca2). The SBS reduction in Zr-c2T, Zr-Ca1T, and Zr-Ca2T relative to their respective non-aged groups was lower than that in Zr-c1T vs. Zr-c1, demonstrating superior bond strength retention following aging. Furthermore, thermocycling did not alter the relative SBS hierarchy: Zr-Ca1T maintained a higher SBS than Zr-c2T, while Zr-Ca2T retained a significantly higher SBS than the other three groups.

**Figure 7:**
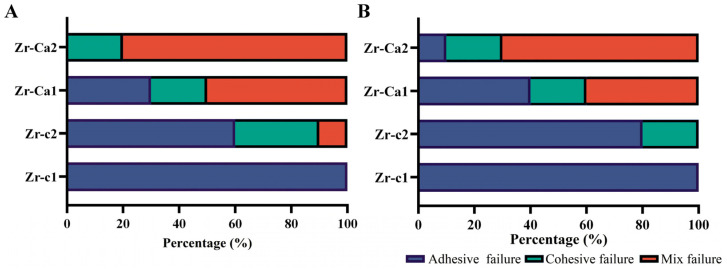
Failure mode distribution of the control and experimental groups before (**A**) and after (**B**) thermocycling. Zr-c1: untreated high-translucency zirconia using resin adhesive; Zr-c2: untreated high-translucency zirconia using resin adhesive containing MDP; Zr-Ca1: surface-generated calcium zirconate particle group using resin adhesive; Zr-Ca2: surface-generated calcium zirconate particle group using resin adhesive containing MDP.

The failure mode distribution is shown in Figure 7. Before and after thermocycling, the failure mode of the Zr-c1 group exhibited adhesive failure. However, in the Zr-c2T group, the fracture mode was still dominated by adhesive fracture, but due to the use of resin adhesives containing MDP, the proportion of adhesive fractures (60%) was reduced, and cohesive fracture and mixed fracture modes appeared. After thermocycling, the proportion of adhesive fracture mode increased, and the proportion of cohesive fractures decreased. In addition, the proportion of adhesive fracture mode further decreased in group Zr-Ca1T, reaching approximately 30%, and the fracture mode was mainly mixed fracture, about 50%. The aging test increased the proportion of adhesive fracture mode in the Zr-Ca1T group and decreased the proportion of mixed fracture mode. The results of the Zr-Ca2 group showed that mixed fracture mode accounted for 80%, cohesive fracture accounted for 20%, and no adhesive fracture mode was found. After the aging test, a small proportion of adhesive fracture mode (10%) appeared.

## 4. Discussion

In this study, we modified the surface of HTZ by synthesizing CaZrO_3_ particles in situ to promote both micromechanical interlocking and chemical bonding. Our results confirmed the successful formation of surface CaZrO_3_ on HTZ and a significant increase in bond strength to resin cement. In addition, pre-treatment with MDP further elevated the shear bond strength and improved resistance to aging. Accordingly, the null hypothesis that in situ-synthesized CaZrO_3_ would have no effect on the bonding performance of HTZ was rejected.

High-translucency zirconia (HTZ) not only improves the optical properties of conventional zirconia but also exhibits superior wear resistance and antibacterial activity compared to glass ceramics, making it an ideal material for minimally invasive restorations [26]. A recent study comparing metal–resin bonded bridges and HTZ–resin bonded bridges in the anterior region demonstrated that, compared with traditional metal–resin bonded bridges, HTZ–resin bonded bridges reduced the retainer thickness under physiological occlusal force—from 0.8 mm to 0.5 mm—thereby minimizing the amount of tooth preparation. Additionally, the 0.5 mm preparation ensures that the adhesive interface is located within the enamel layer, which guarantees the bonding strength between the adhesive and dental hard tissues [27]. This study highlights the potential application value of HTZ as a minimally invasive restorative material in the anterior dental region. However, to achieve long-term functional and esthetic stability after restoration, HTZ-based minimally invasive restorations must possess favorable resin bonding strength. Similar to other brittle ceramic materials, robust resin bonding not only provides adhesion between the ceramic restoration and the tooth but also enhances the flexural strength of the restoration. Currently, the evidence-based bonding protocol for zirconia with sufficient clinical evidence is the “APC zirconia bonding concept”, which consists of three key steps: airborne particle abrasion (APA) using alumina or silica-coated alumina particles (Step A), primer application with MDP (10-methacryloyloxydecyl dihydrogen phosphate) or phosphate monomer-based primers on the abraded surface (Step P), and bonding with dual-cure or self-cure composite resin cements (Step C) [28]. Airborne particle abrasion primarily increases the surface roughness of the restoration, thereby expanding the bonding area. The application of MDP phosphate monomers can hydrolyze the dentin surface to form Ca-P bonds, strengthening the chemical adhesion. Nevertheless, HTZ has enhanced yttrium oxide (Y_2_O_3_) content, which impairs its phase transformation toughening capacity and thus limits its mechanical strength. Moreover, post-sintering APA may exert adverse effects on the HTZ surface due to variations in abrasive particle selection, particle size, air pressure, abrasion time, angle, and distance [29]. Furthermore, the long-term durability of the aged bond of HTZ treated with this protocol remains unclear [30].

In recent years, several studies have recommended performing surface treatments in the Zirconia pre-sintering stage and have reported multiple advantages [31,32,33,34,35,36,37]: (a) the higher porosity and lower chemical stability before sintering allow more effective roughening, thereby improving micromechanical interlocking with resin cement; (b) sintering after treatment blunts sharp defects and mitigates crack precursors; and (c) sintering reduces the monoclinic phase introduced by surface treatments. Building on this rationale, our previous work generated in situ ZrO_2_ particles on conventional pre-sintered zirconia by sequential immersion in hydrofluoric acid, calcium chloride, and sodium hydroxide solutions, which improved bonding to resin/porcelain primarily through enhanced mechanical interlocking [21]. This method avoids the potential adverse effects of post-sintering surface air abrasion on the material’s inherent mechanical strength, and preliminary studies have confirmed that the formation of ZrO_2_ particles on the surface does not compromise the material’s mechanical properties. Furthermore, based on the “APC principle”, in order to further increase the surface chemical reactivity of high-translucency zirconia, we adjusted the process to introduce reactive Ca^2+^ and to form CaZrO_3_ particles on the material surface.

The reactions underlying this approach are summarized in Equations (3)–(7). Immersion of pre-sintered zirconia in NaF and HCl yields ZrF_4_, which reacts with CaCl_2_ to produce ZrOCl_2_. Hydrothermal treatment with NaOH converts ZrOCl_2_ to zirconium hydroxide, Zr(OH)_4_, while CaCl_2_ reacts with NaOH to form Ca(OH)_2_. Finally, high-temperature calcination at 1450 °C drives the solid-state reaction of Zr(OH)_4_ with Ca(OH)_2_ to generate CaZrO_3_, thereby synthesizing CaZrO_3_ particles in situ on the HTZ surface.

Chemical equations used in the present study were as follows:

ZrO_2_ + 4NaF + 4HCl → ZrF_4_ + 4NaCl + 2H_2_O (3)

ZrF_4_ + 2CaCl_2_ + H_2_O → ZrOCl_2_ ↓ + 2CaF_2_ ↓ + 2HCl (4)

ZrOCl_2_ + 2NaOH + H_2_O → Zr(OH)_4_ ↓ + 2NaCl (5)

CaCl_2_+ 2NaOH → Ca(OH)_2_ ↓ + 2NaCl (6)

Zr(OH)_4_ + Ca(OH)_2_ → CaZrO_3_ + 3H_2_O ↓ (7)

The purpose of this study is to investigate the hydrolysis of surface-generated calcium (Ca) upon its interaction with MDP, which facilitates the formation of Ca-P bonds. This not only enhances mechanical interlocking between resin and the high-translucency zirconia (HTZ) surface but also strengthens chemical adhesion, thereby effectively improving the immediate bonding performance of HTZ and endowing it with excellent aging resistance.

Since excessive deep acid etching can lead to a decrease in the flexural strength of the entire material, we adopted the method of slowly adding HCl to NaF solution to effectively control the acid etching depth on the HTZ surface. Ideally, we expect the acid etching depth to match the Ca^2+^ penetration depth. To effectively ensure the depth and progress of this reaction, glycerol was incorporated to modulate viscosity and thereby regulate the diffusion and penetration of Ca^2+^. The glycerol volatilized during high-temperature sintering without affecting CaZrO_3_ synthesis. We repeatedly tested the concentration ratio through preliminary experiments and have now achieved the ideal effect. Three-point bending strength decreased slightly after treatment (from 834.12 to 757.23 MPa). Although the strength after treatment is slightly reduced at present, it is still higher than that of glass ceramics [2,38,39,40]. The current flexural strength can effectively meet the needs of clinical applications. The XRD pattern showed tetragonal and cubic reflections at 24.2°, 28.3°, 31.5°, 34.4°, 35.4°, and 51.3°, corresponding to the (110), (−111), (111), (002), (200), and (220) planes, respectively [41]. Most of these reflections were also observed in Zr-c, indicating that the crystallinity of HTZ was largely preserved, with only a small amount of monoclinic phase present (Figure 4A) [42]. SEM and WLI revealed regularly shaped particulate features on the Zr-Ca surface (Figure 2A,C). EDS confirmed that the particulates comprised zirconium, oxygen, and calcium (Table 1). In the study, whether the calcium zirconate formed on the surface and the resin adhesive containing phospholipids truly exert the effects of enhancing chemical adhesion has been further verified through mechanistic validation experiments. XPS showed characteristic signals attributable to calcium, zirconium, and oxygen with spectral features consistent with Ca-O and Zr-O bonding, indicating successful CaZrO_3_ formation on the Zr-Ca group (Figure 4C). FTIR likewise displayed bands assignable to Ca-O and Zr-O vibrations, further supporting the formation of CaZrO_3_ (Figure 4B). Overall, these results confirm that CaZrO_3_ particulates can be synthesized in situ on the HTZ surface while maintaining mechanical properties within clinically acceptable limits. In immediate shear bond strength testing, the SBS of the Zr-Ca group was significantly higher than that of the control, indicating that in situ-synthesized CaZrO_3_ particulates markedly improved the bonding properties of HTZ. The CaZrO_3_ particulates increased surface roughness, enlarging the effective bonding area and introducing topographic irregularities that promote stronger micromechanical interlocking relative to the smooth, polished HTZ substrate (Figure 3A). These results are consistent with our previous investigations on in situ-synthesized ZrO_2_ particulates on conventional zirconia [21,43], and they highlight the central role of surface roughening in promoting zirconia–resin bonding. Moreover, water contact angles on Zr-Ca specimens were significantly lower than those on HTZ, indicating that in situ-synthesized CaZrO_3_ increased the surface hydrophilicity of HTZ and facilitated adhesive spreading on the material surface.

The application of primers containing MDP is well known to improve the bonding performance of zirconia ceramics by promoting the formation of Zr-O-P bonds, thereby contributing to long-term durability under clinical conditions [44,45,46,47,48]. However, this coordination bond is prone to hydrolysis and aging. Therefore, general studies have shown that the 10-MDP bonding system can improve the immediate bonding strength of zirconia ceramics, but the improvement of bonding strength after aging is not ideal. A long-term stable bonding effect is the key to enabling zirconia-based restorations to achieve the functional outcomes of minimally invasive esthetic rehabilitation. As shown in Figure 6, the immediate bonding strength was markedly enhanced when conventional high-transparency zirconia was treated with the 10-MDP-based resin adhesive (Zr-c2). Nevertheless, a significant decline in bonding strength was still observed following thermocycling, which verified the experimental phenomenon identified in the preliminary work. In contrast, for high-transparency zirconia with a surface modified via the protocol established in this study and bonded with the same 10-MDP resin adhesive (Zr-Ca2), the immediate bonding strength was further significantly elevated compared with the Zr-c2 group. More importantly, the bonding strength was well retained after thermocycling treatment, and the residual bonding strength of the Zr-Ca2 group was approximately twice that of the Zr-c2 group. The underlying mechanism for this improved bonding performance is as follows. In comparison with the intrinsic ZrO_2_ phase, the formation of CaZrO_3_ particles enhances the surface roughness, thereby augmenting mechanical interlocking with the bonding resin. Meanwhile, the in situ-formed CaZrO_3_ particles are anticipated to possess a higher density and a broader distribution of interfacial metal active sites (associated with Ca and Zr ions), which can form effective interactions with the phosphate groups of 10-MDP. The chemical interaction between Ca^2+^ and phosphate groups enables the formation of relatively stable Ca-P covalent bonds, which mitigates the drawback of facile dissociation associated with simple hydrogen bonding interactions [49,50]. This dual-site chemical reaction is also consistent with the well-documented tendency of 10-MDP to form stable calcium salts and layered structures in calcium-rich mineral matrices. Collectively, these results suggest that the introduction of interfacial Ca^2+^ is conducive to enhancing the long-term bonding stability between zirconia and 10-MDP-based resin adhesives. In addition, Zr-Ca2 exhibited a further increase in SBS compared with both Zr-Ca1 and Zr-c2. These findings suggest a stronger bond and an additional chemical interaction between MDP and CaZrO_3_, supported by the detection of Ca-P bonds in XPS (Figure 4(Ci)) and FTIR (Figure 4B). Although FTIR is not quantitative, the spectra substantiate that a reaction between CaZrO_3_ and MDP occurred, and the introduction of calcium on the HTZ surface imparted greater chemical reactivity. A total of 5000 thermocycles were applied to challenge the durability of the resin composite bonded to HTZ. After thermocycling, SBS decreased significantly for Zr-c1, Zr-c2, and Zr-Ca1, indicating sensitivity to the aging process (Figure 6). Nevertheless, the SBS values of Zr-c2, Zr-Ca1, and Zr-Ca2 remained significantly higher than those of the untreated group, with Zr-Ca2 retaining the highest SBS after thermocycling. This performance likely reflects the combined effects of micromechanical interlocking from CaZrO_3_ particulates and chemical bonding arising from the MDP-Ca linkage. Accordingly, within the limits of this study, neither in situ-synthesized CaZrO_3_ particulates nor MDP treatment alone was sufficient to resist aging, whereas their combination appears promising for improving both bond strength and durability of high-translucency zirconia. Finally, the results of fracture mode analysis also demonstrated that the modification method for generating CaZrO_3_ particulates on the high-translucency zirconia surface enhanced the covalent interaction with the 10-MDP resin adhesive. Mixed failure predominated in the Zr-Ca1 (50%) and Zr-Ca2 (80%) groups, whereas adhesive failure predominated in the Zr-c2 (60%) group; Zr-c1 exhibited exclusively adhesive failures, indicating that the presence of in situ-synthesized CaZrO_3_ on the HTZ surface strengthens the interfacial connection. Notably, the proportion of mixed failure in Zr-Ca2 was the highest, which showed 80% before aging and still 70% after aging (Figure 6). These failure patterns support the formation of chemical bonding between zirconia and resin cement under the combined action of CaZrO_3_ and MDP, effectively improving interfacial quality. This is consistent with previous observations in dentin bonding, where nanolayered MDP-Ca salts at the dentin–adhesive interface exhibit a higher elastic modulus than the surrounding adhesive and form three-dimensional continuous structures that mechanically reinforce the adhesive in a manner analogous to filler particles [51].

In addition to the functional aspects discussed above, the formation of CaZrO_3_ particulates and the accompanying surface changes could, in principle, affect the optical appearance of 3Y-TZP by modifying light scattering near the surface. However, in clinical practice, the treated layer serves as the bonding surface and may be partially altered by finishing/polishing and subsequently covered by luting cement and restorative materials, so any net change in perceived translucency may be limited. However, a systematic evaluation using established dental optical metrics will be performed in future work.

## 5. Conclusions

Within the limitations of this study, we show that CaZrO_3_ particulates can be formed in situ on the surface of high-translucency zirconia (HTZ) by leveraging the higher porosity and lower chemical stability of the pre-sintered substrate. This in situ modification increased surface roughness and wettability, significantly enhancing the immediate bond strength of HTZ to resin cement and conferring partial resistance to artificial aging. Bond strength was further improved when the in situ CaZrO_3_ particulates were combined with MDP-containing primers, with the effect most evident after aging. The treated HTZ maintained flexural strength within clinically acceptable limits and showed no detectable surface phase transformation, suggesting no predisposition to early degradation. Collectively, these findings indicate that in situ CaZrO_3_ formation is a promising approach to achieving strong, durable resin–zirconia bonding in clinical practice; nonetheless, further work is needed to optimize application parameters and to verify long-term in vivo performance.

## Figures and Tables

**Table 1:** The atomic content ratio of each group of samples.

Content of Different Element (Atom %)			
Group	Zr	O	Ca
Zr-c	40.59%	59.41%	0
Zr-Ca	34.84%	62.05%	3.11%

## Data Availability

The original contributions presented in this study are included in the article. Further inquiries can be directed to the corresponding authors.

## Supplementary Materials

The following supporting information can be downloaded at: https://www.mdpi.com/article/10.3390/jfb17050227/s1, Figure S1: The arithmetic mean height of the 3D surface roughness of control and modification groups.
