# Supplementary figures and images for: In Situ Formation of Calcium Zirconate Particles on the Surface of High-Translucent Zirconia: A New Way to Strongly Improve Its Bonding Properties
Source: J Funct Biomater. 2026 May 6;17(5):227. doi: 10.3390/jfb17050227 (PMC13207787; doi:10.3390/jfb17050227)

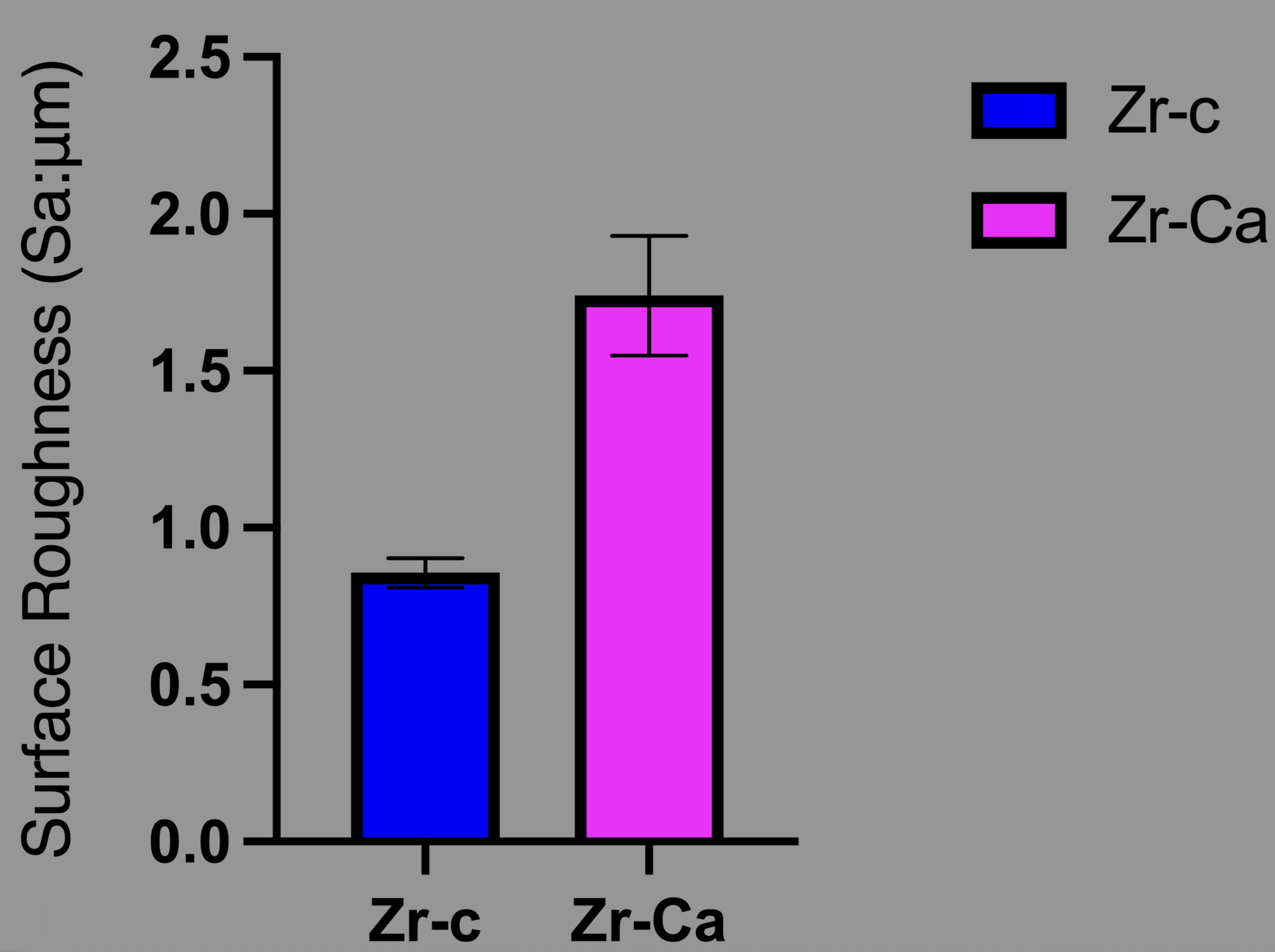

Supplement: Supplementary file 1 [file jfb-17-00227-s001.zip › jfb-4104652-supplementary.png]
